# Supplementary material for: OpticalFlow3D – a tool for measuring amorphous motion in three-dimensional fluorescence microscopy images
Source: J Cell Sci. 2026 Jul 15;139(21):jcs264851. doi: 10.1242/jcs.264851 (PMC13405222; doi:10.1242/jcs.264851)
Supplement: Supplementary information [file joces-139-264851-s1.pdf]

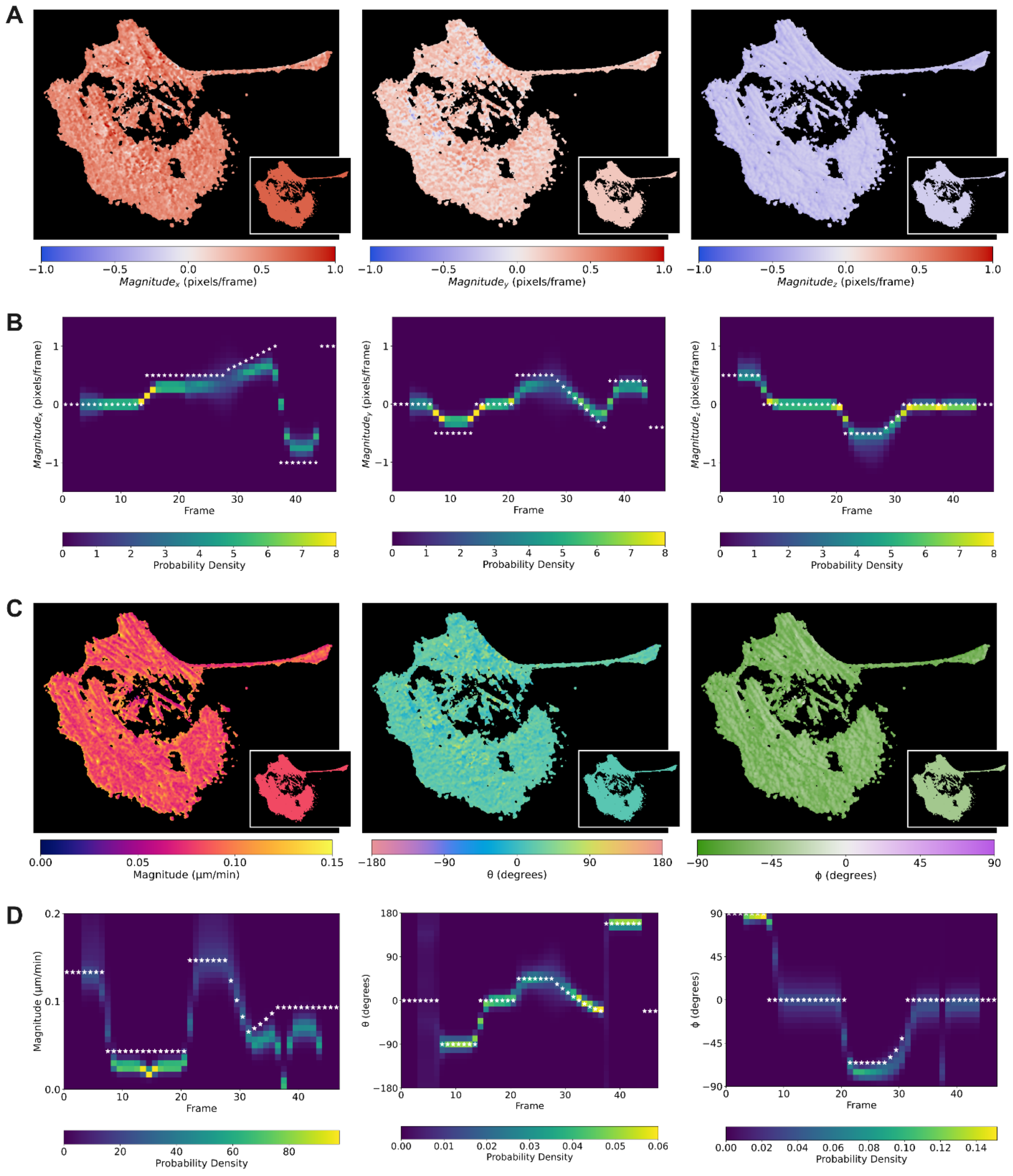

Fig. S1. Optical flow is not equivalent to translation. Individual components of the optical flow field are shown for an image (frame 1 of the cell in Fig. 1) that was artificially translated over time (A). Insets indicate the expected image if optical flow was equivalent to translation. Over time, optical flow tends to underestimate the expected translational motion, which is indicated by white stars (B). Flow is less accurate for magnitude than for measurements of direction (ϑ and φ) as shown by the calculated flow fields and the translation inset (C). This is reflected in measures of magnitude and direction over time (D), where translational motion is indicated by white stars. Figures created with Python implementation using a 90th percentile reliability threshold.

Journal of Cell Science • Supplementary information

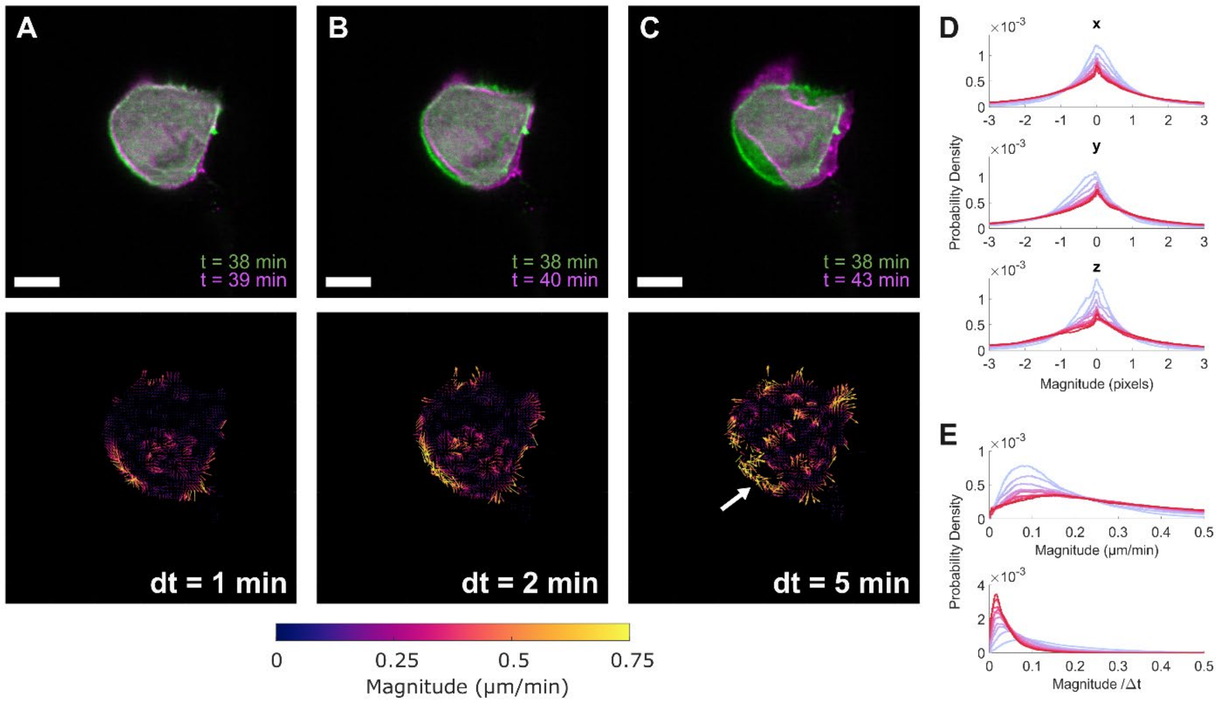

Fig. S2. Frame rates should be sufficiently fast to have largely subpixel motion. When the time between frames is small (A), optical flow (bottom) reflects the motion seen in the images (top). As the time between frames increases, the optical flow becomes noisier (B). When the time between frames is too large (C), optical flow is noisy, does not reflect the motion seen in the image well, and regions begin to be dropped due to low reliability (gap near arrow). Optical flow is ideally suited to subpixel movement, and as the time between frames increases, this assumption is increasingly violated (D). As the time between frames increases, a rough approximation is that the magnitude of motion should increase as well (E, top). However, it does not increase proportionally to dt (E, bottom), indicating that the flow is likely missing a portion of the motion. Images shown are a single slice from the lattice light sheet data shown in Figure 5. Figures were created with the MATLAB implementation using the 92nd percentile of reliability as a threshold. Scale bars are 10 μm.

Journal of Cell Science • Supplementary information

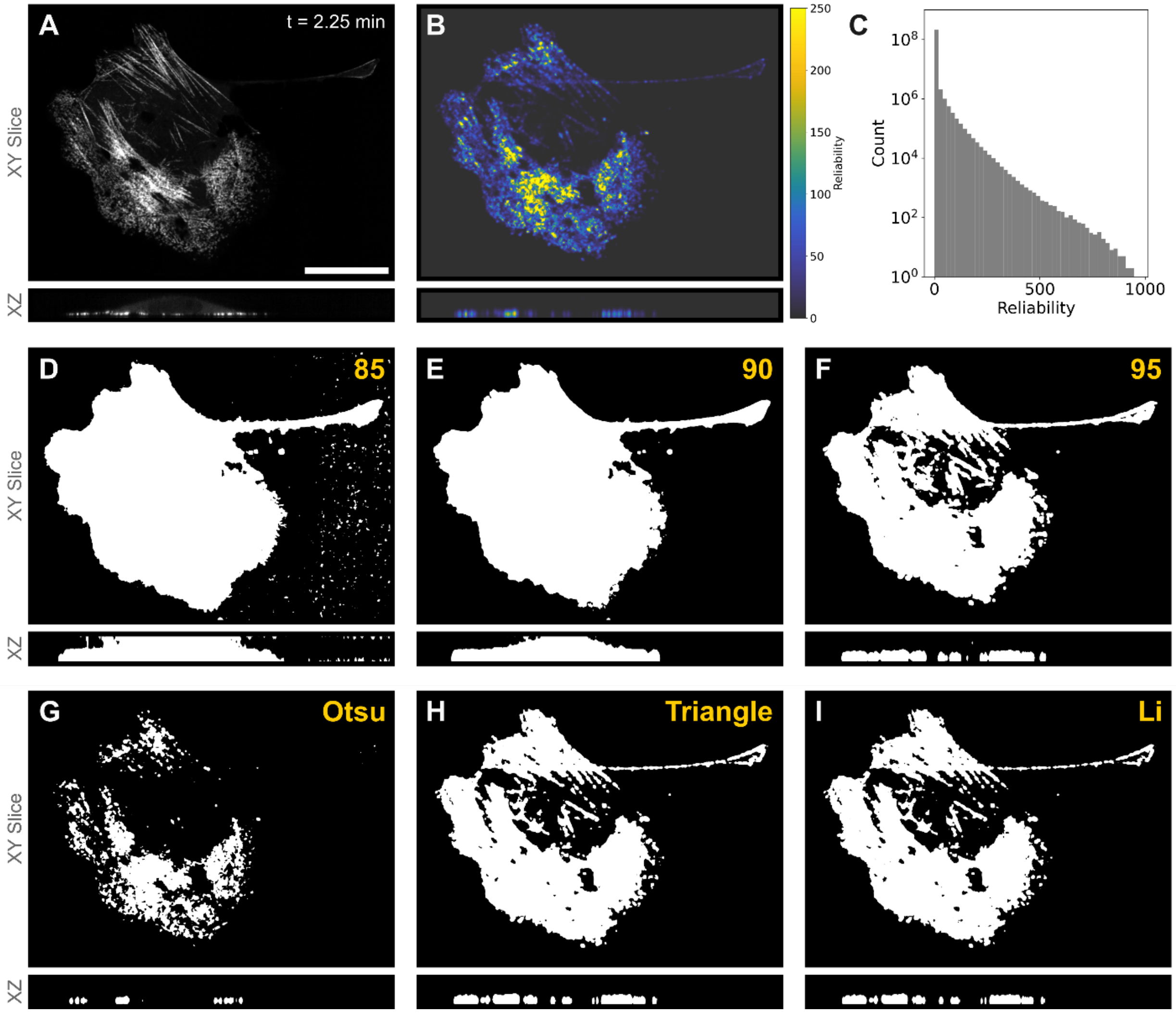

Fig. S3. Reliability can be used as a threshold on optical flow. A single xy-slice and xz-slice from spinning disk confocal imaging of myosin II (A, also shown in Fig. 1) lead to high reliability values in regions with clear gradients (B). The distribution of reliability values includes a peak near zero for background noise (C). Setting a threshold on reliability allows for removal of spurious components (D-F). Using the 85th percentile of reliability results in inclusion of spurious noise (D), while the 90th percentile provides a clean segmentation of the cell (E). Higher thresholds can be selected (95th percentile, F) to emphasize flow confidence at the expense of some cellular regions. A variety of automatic threshold algorithms could be appropriate depending on the segmentation goal. An Otsu threshold (G) highlights only the brightest regions of myosin II, while the Triangle (H) and Li (I) algorithms accept dimmer regions of the cell. The Triangle and Li algorithms highlight similar features in this case; selection of an algorithm should consider the biological system being imaged. Thresholds should be held consistent across replicates and conditions for a fair comparison. Scale bar is 25 µm. Figure created with the Python implementation. This cell is also shown in Figs 1, 2, S1, S7 and S8.

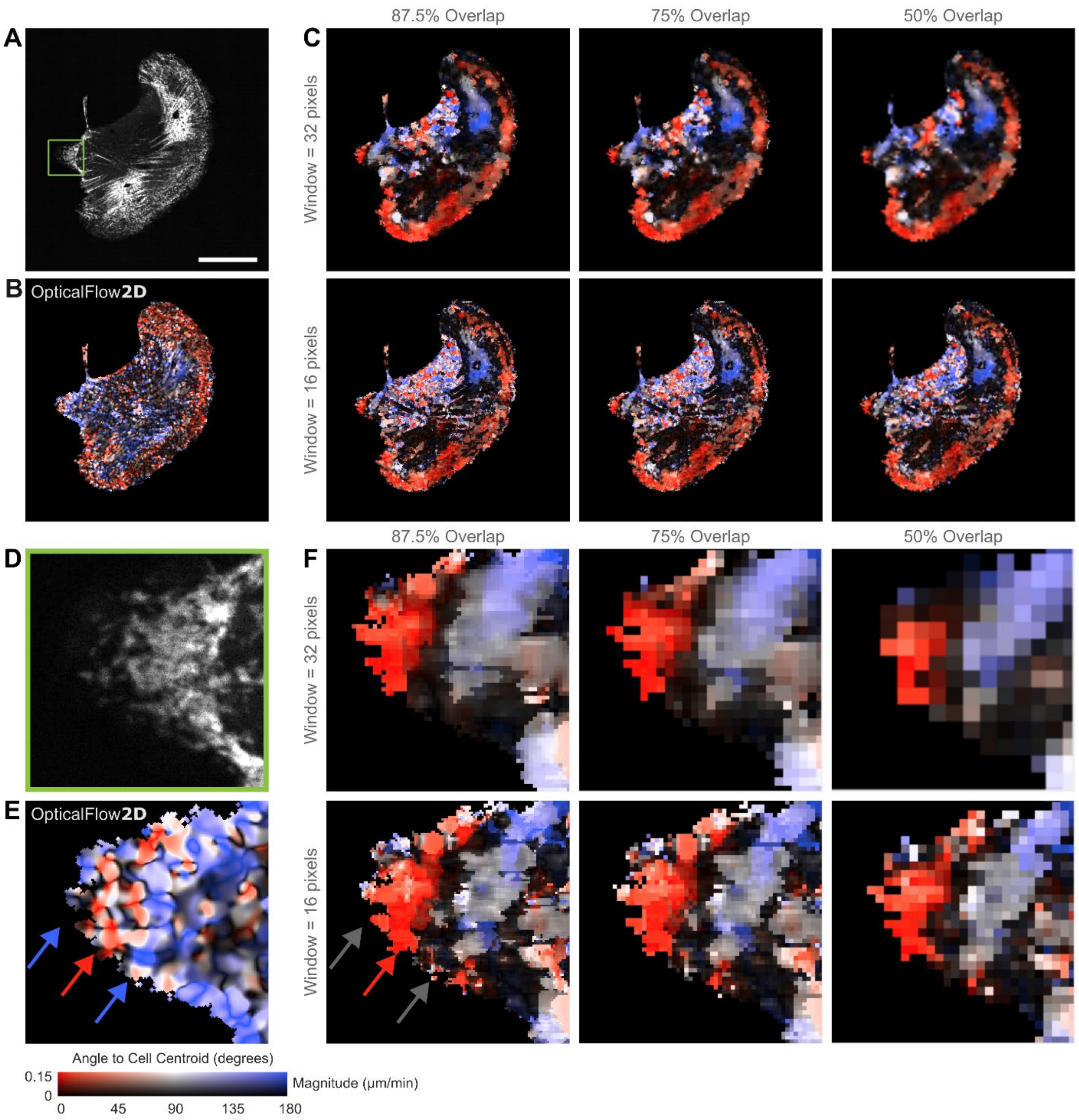

Fig. S4. PIV captures motion on the scale of the interrogation window. Z-slice from Fig. 3 shown at t=15 min (A). Scale bar is 25 μm. 2D optical flow on this z-slice captures the extension of the leading edge (B), in agreement with 3D calculations (Fig. 3). Snapshots of 2D particle image velocimetry (PIV) are shown across two interrogation window sizes (16 or 32 pixels) and three overlaps (50%, 75%, 87.5%) in (C). A small ROI (D, green box in A) highlights the dense flow field from optical flow (E, Fig. S5D). PIV flow fields (F, Fig. S5E,F) are pixelated due to interrogation window size and window overlap. Although increasing the % overlap increases the sampling density, the motion detected by PIV is still limited to motion occurring on the scale of the interrogation window. In regions where opposing motion occurs on smaller length scales, PIV averages over the motion and reports low velocities (dark regions in C, F). Note that even at the highest sampling density, PIV is unable to simultaneously capture protrusion extension (E, blue arrow) and formation of a myosin II contractile zone (E and F, red arrow). Gray arrows indicate missing regions of extension in the PIV flow field.

Journal of Cell Science • Supplementary information

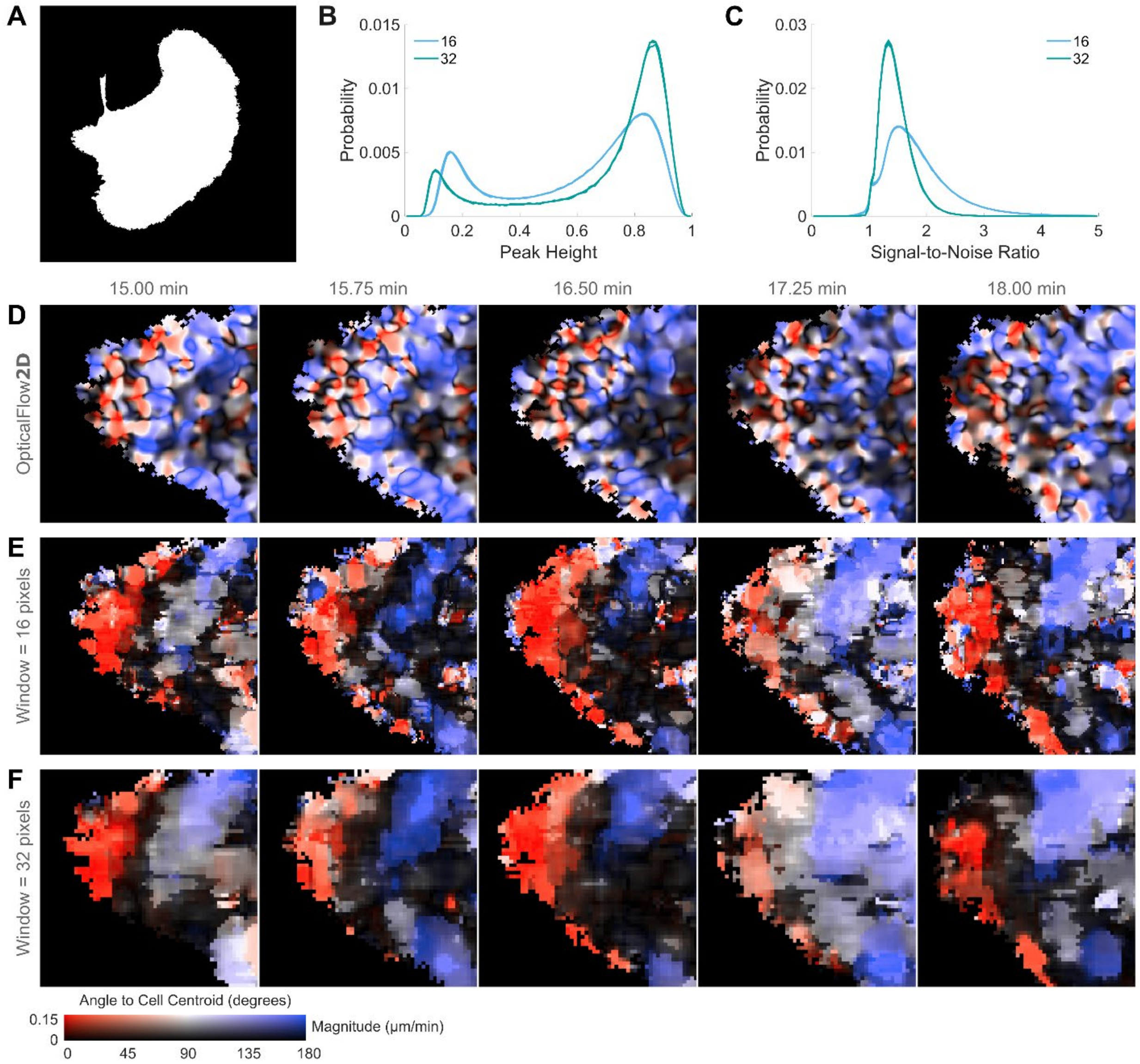

Fig. S5. PIV quality depends on interrogation window size. For a consistent comparison, PIV and optical flow fields were masked by the same cell segmentation. The original intensity image was segmented using 40% of the Otsu threshold at each frame, after which the binary image was dilated by a disk of radius two pixels and binary holes were filled. The largest remaining object (A) was used as a mask for the flow fields. Correlation peak height (B) and signal-to-noise ratio (SNR, C) reflect the quality of the PIV calculations. SNR is calculated as the height of the largest correlation peak divided by the height of the second largest peak. Each window size shows three overlapping curves, corresponding to 50, 75, and 87.5% window overlap. Overlap does not impact peak height or SNR. Increasing the interrogation window size from 16 to 32 pixels leads to larger (more robust) peak heights (C). SNR values remain low at 32 pixels; at both window sizes the number of features within a window is limited, decreasing overall quality. This uncertainty is reflected in stability over time (D-F). OpticalFlow2D exhibits smoothly evolving flow fields (D). PIV results are shown at 87.5% overlap for 16 (E) and 32 (F) pixel interrogation window sizes. Both E and F exhibit less consistent flows over time compared to optical flow, especially in regions of the cell (Fig. S4D) that do not exhibit multiple discrete features within a single interrogation window. This analysis is of the same cell as shown in Figs 3 and S4.

Journal of Cell Science • Supplementary information

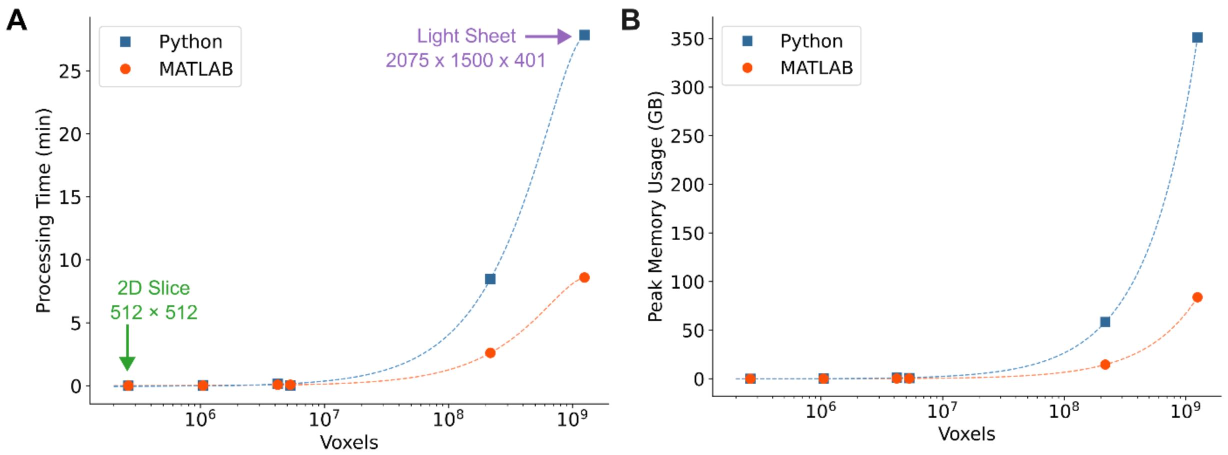

Fig. S6. Implementation performance for a single time point. A single time point of images of different sizes (total voxels, x-axis) was analyzed ten times with each implementation on a workstation PC (see Methods). The average processing time for the ten replicates is shown in (A). The smallest image analyzed was a 2D slice with size 512 pixels by 512 pixels, while the largest example is a light sheet acquisition of size 2075 by 1500 by 401 pixels. The maximum memory usage across the ten replicates is shown in (B). The Python implementation requires more time and memory for large datasets.

Table S1. Processing Time and Memory Usage

Image Size (x × y × z)	Voxels	MATLAB (Workstation)		MATLAB (Laptop)		Python (Workstation)		Python (Laptop)	
512 × 512 × 1	2.6×10^5	0.627 s	0.01 GB	2.45 s	0.01 GB	0.164 s	0.03 GB	0.707 s	0.03 GB
1024 × 1024 × 1	1.0×10^6	0.959 s	0.06 GB	4.49 s	0.06 GB	0.510 s	0.13 GB	2.01 s	0.13 GB
512 × 512 × 4	1.0×10^6	1.09 s	0.07 GB	4.47 s	0.07 GB	2.61 s	0.28 GB	5.65 s	0.28 GB
1024 × 1024 × 4	4.2×10^6	5.07 s	0.28 GB	15.5 s	0.28 GB	9.91 s	1.13 GB	20.4 s	1.13 GB
2304 × 2304 × 1	5.3×10^6	4.86 s	0.28 GB	17.0 s	0.28 GB	1.90 s	0.70 GB	9.40 s	0.70 GB
2304 × 2304 × 41	2.2×10^8	156 s	15 GB	Insufficient Laptop Memory		508 s	58 GB	Insufficient Laptop Memory	
2075 × 1500 × 401	1.2×10^9	516 s	84 GB	Insufficient Laptop Memory		1670 s	351 GB	Insufficient Laptop Memory	

Journal of Cell Science • Supplementary information

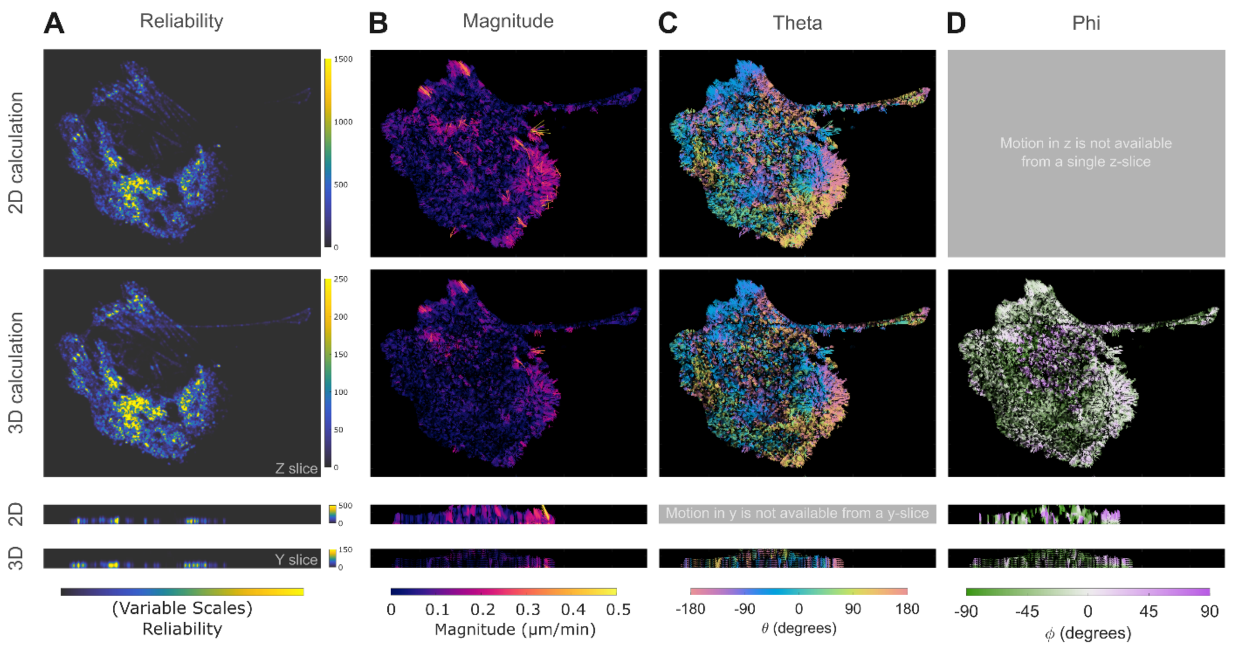

Fig. S7. Comparison of 2D and 3D optical flow. The upper row indicates flow results from analyzing an isolated xy plane (i.e., a 2D image series) from the full 3D image sequence, while the second row indicates the results on that same plane but from analysis of the full 3D volume. The third row indicates a single xz slice extracted and analyzed as a 2D image sequence, while the fourth and final row indicates the results for that same slice from analysis of the full volume. Reliability values (A) have similar patterns across 2D and 3D, although reliability is higher on the trailing protrusion in 3D as it is a thin structure in 2D, but a more complete object in 3D. Note that the reliability scales across each image are different; only relative, rather than absolute values of this metric are useful for thresholding. The 2D and 3D approaches also reach similar results for magnitude (B), ϑ (C), and φ (D), although the 2D results are generally noisier. The 3D approach can better account for out-of-plane motion and includes an additional smoothing in z that removes part of the noise seen in 2D. This figure was generated from the MATLAB implementation for a better comparison to the previously published 2D MATLAB implementation (Lee et al., 2020), in contrast to images of the same cell shown in Figs 1, 2, and S3, which use the Python implementation. MATLAB analysis of this cell is also shown in Fig. S8. This figure uses a 90th percentile reliability threshold applied to the full 3D analysis. The 3D reliability mask was applied to the 2D images for comparison of the same regions.

Journal of Cell Science • Supplementary information

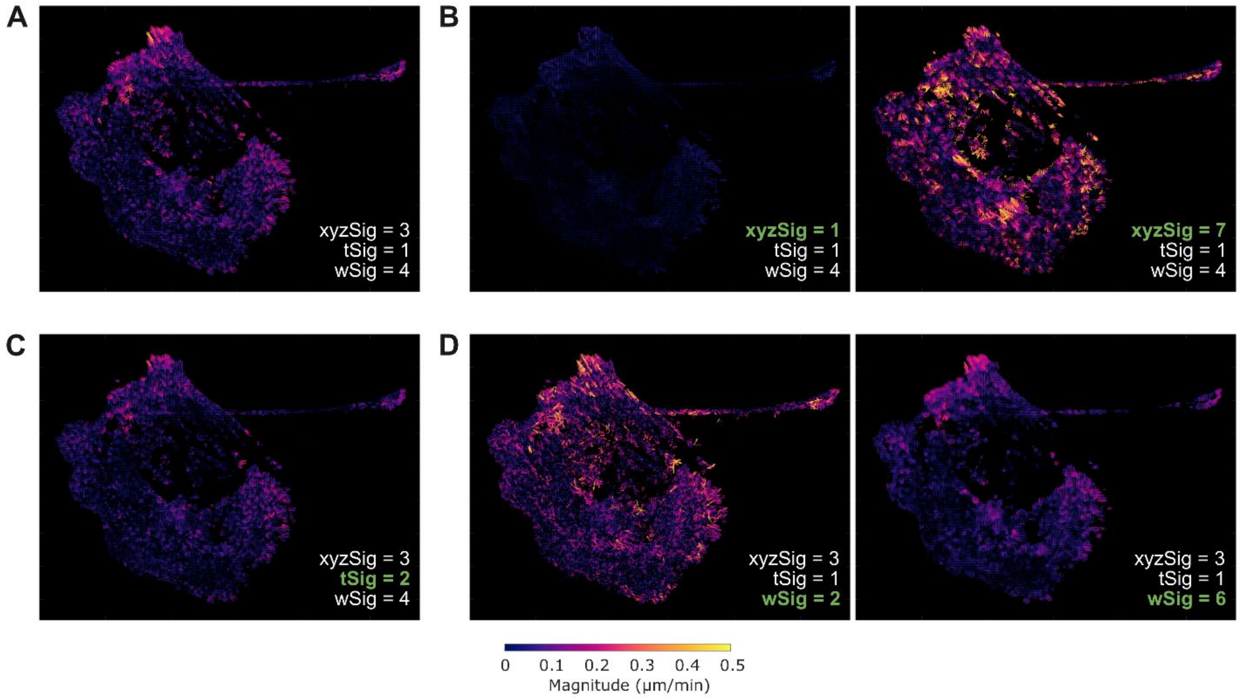

Fig. S8. Optical flow parameters control smoothing. Figures in the main text use xyzSig = 3, tSig = 1, and wSig = 4 (A). Spatial smoothing is controlled by xyzSig (B). When this value is too low, almost no flow is detected as the calculations are dominated by noise. When the smoothing is too high smaller features are removed, and motion tends to be overestimated. Temporal smoothing is controlled by tSig (C). When this value is increased, smaller features are smoothed over. The size of the neighborhood used for the Lucas-Kanade constraint is controlled by wSig (D). When this value is low, the flow field is noisy, but when it is high, small features are smoothed out. Figures from the MATLAB implementation using a 90th percentile reliability threshold. This analysis is of the same cell as shown in Figs 1, 2, S1, S3 and S7.

Journal of Cell Science • Supplementary information

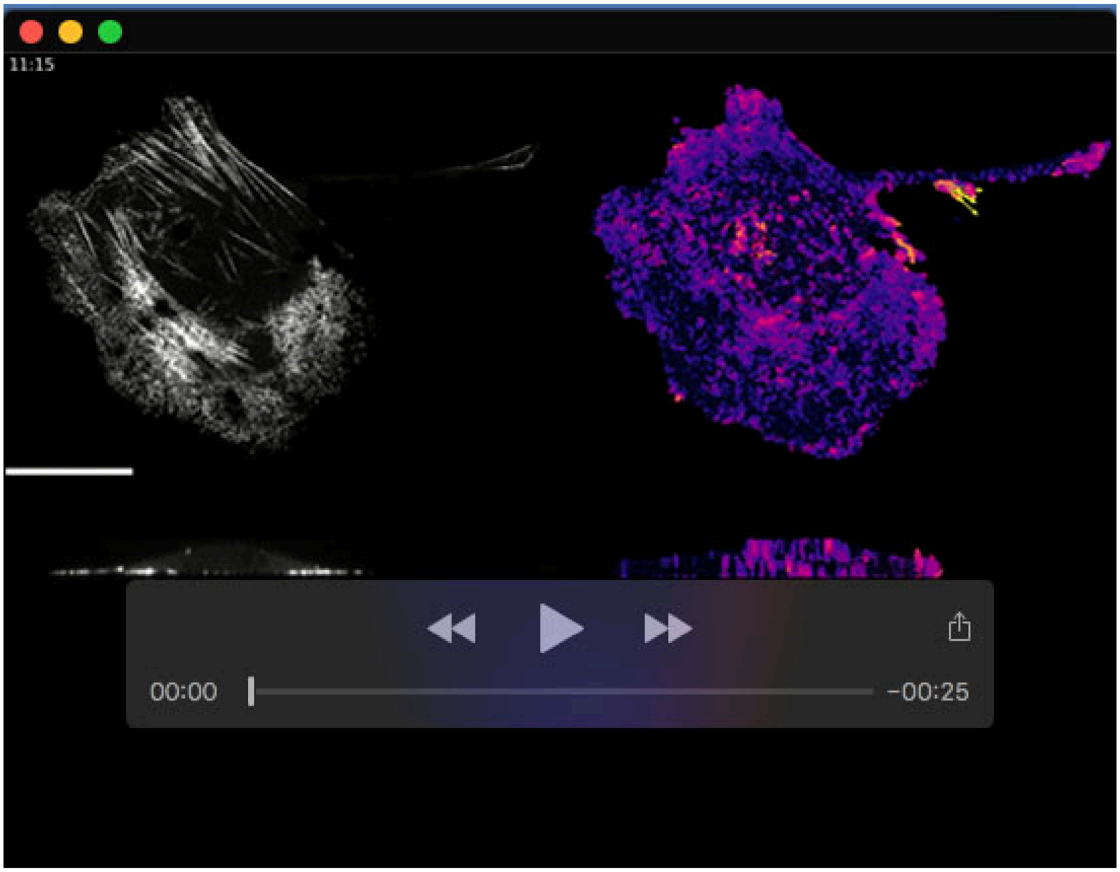

Movie 1. The top portion of the movie shows a single slice in the xy-plane, while the bottom portion shows a single xz-slice. Spinning disk confocal images of myosin II are shown on the left, while the corresponding flow field colored by flow magnitude is shown on the right. The color code for magnitude is as shown in Figure 1 and ranges from 0 (dark blue) to 0.25 (yellow) µm min^{-1}. Images were collected every 45 seconds and are played back at 6 frames per second. The time stamp shows MM:SS and scale bar is 25 µm.

Journal of Cell Science • Supplementary information

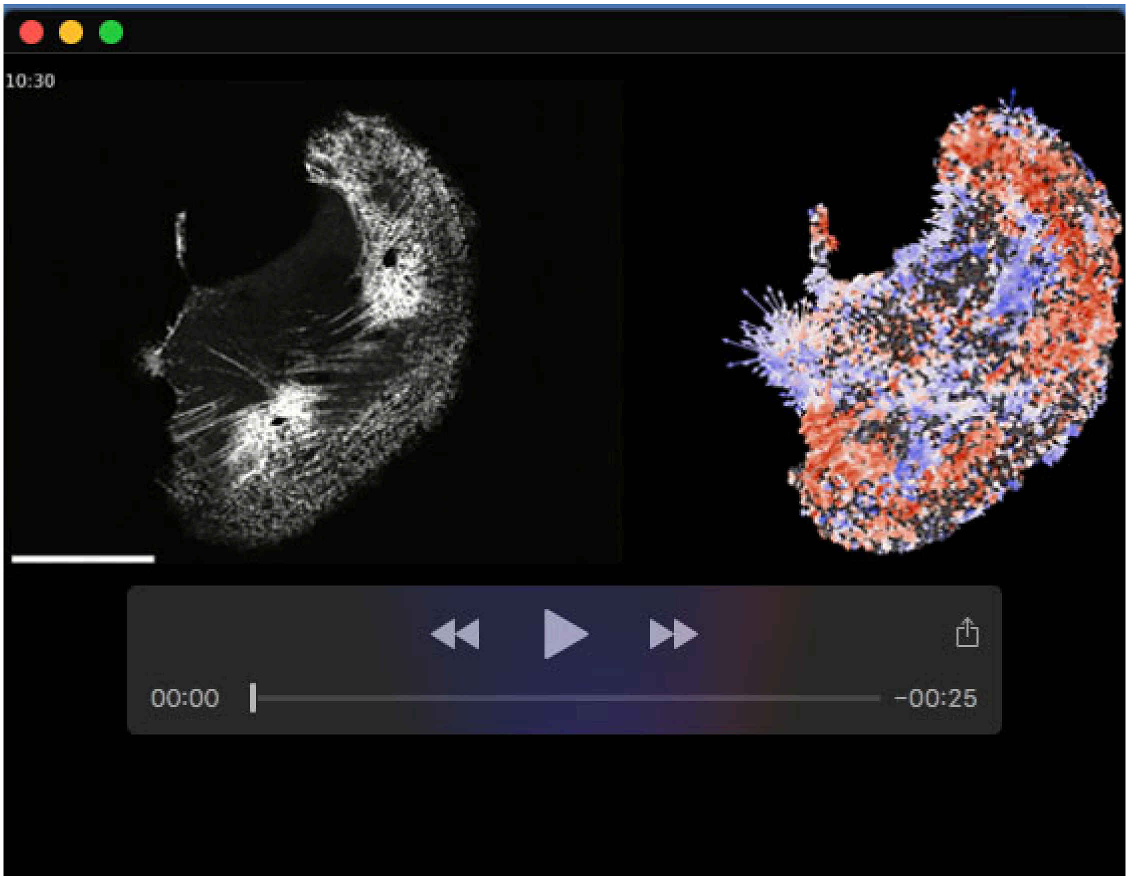

Movie 2. Spinning disk confocal images of myosin II (single xy-plane) are shown on the left, while the corresponding flow field colored by direction with respect to the cell centroid (red = 0 degrees = inward, blue = 180 degrees = outward) is shown on the right. Images were collected every 45 seconds and are played back at 6 frames per second. The time stamp shows MM:SS and scale bar is 25 μm.

Journal of Cell Science • Supplementary information

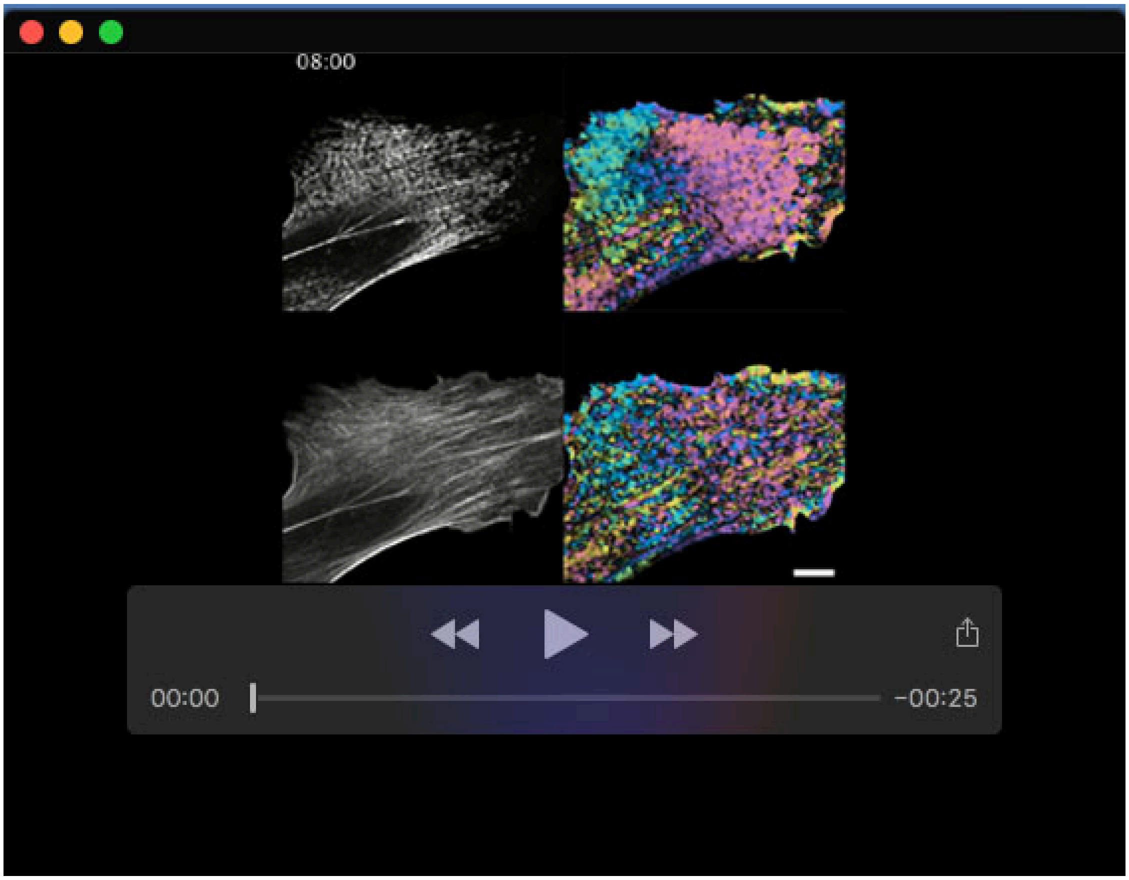

Movie 3. Airyscan confocal images (left) compared to flow fields colored by theta (right) for myosin II (top) and actin (bottom). The color code for theta is as shown in Figure 4. Images were collected every 30 seconds and are played back at 6 frames per second. The time stamp shows MM:SS and scale bar is 5 μm.

Journal of Cell Science • Supplementary information

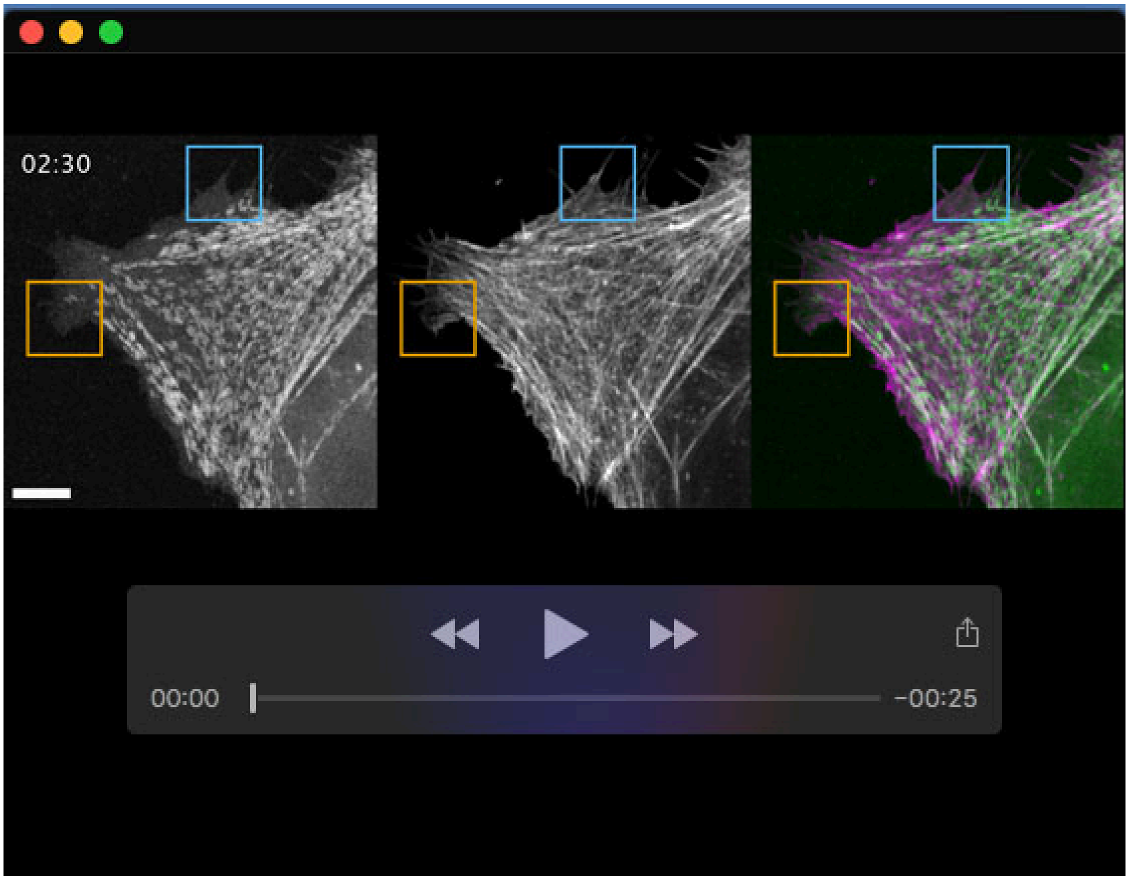

Movie 4. Airyscan confocal images of myosin II (left) and actin (middle) are merged in the right panel (myosin II in green and actin in magenta). ROIs are analyzed separately in Figure 4. Images were collected every 30 seconds and are played back at 6 frames per second. The time stamp shows MM:SS and scale bar is 5 µm.

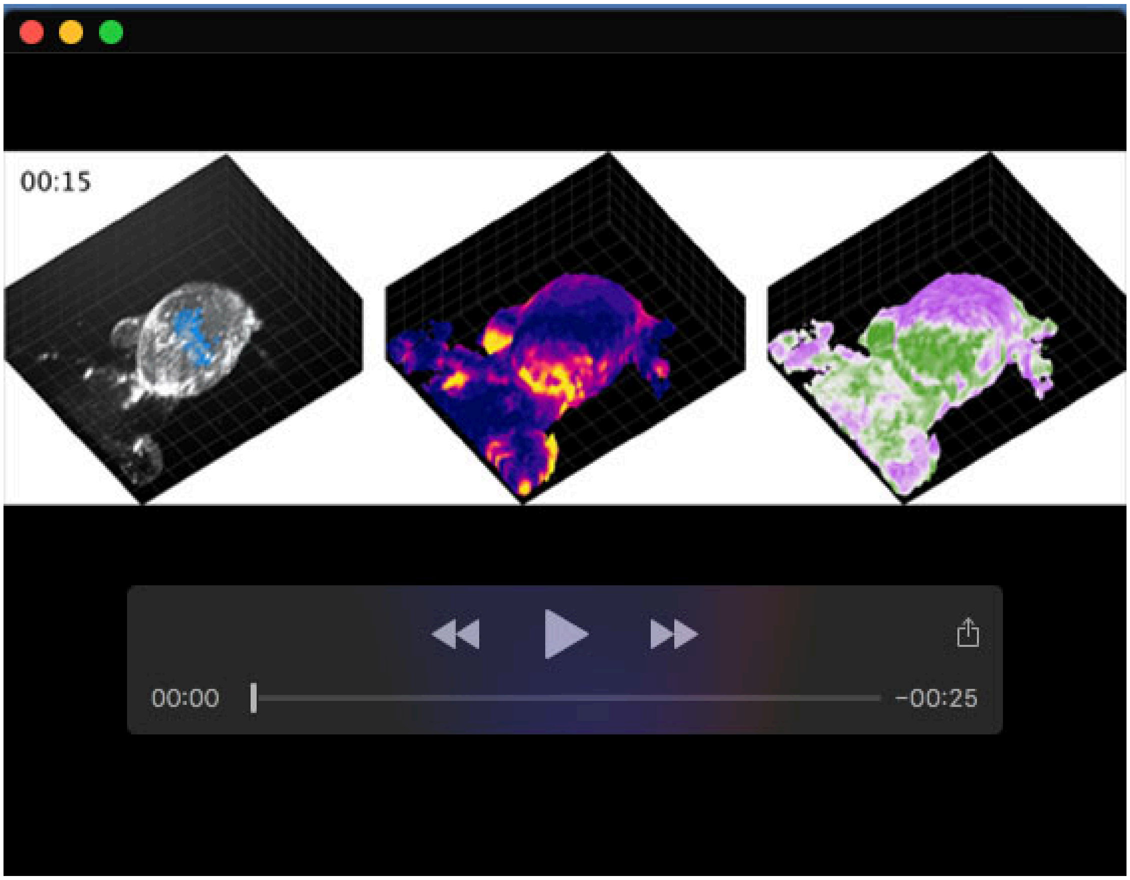

Movie 5. A cropped region of a light sheet image of a dividing cell (left) labeled with Halo-LifeAct (white) and mApple-H2B (blue) is compared to the optical flow magnitude (middle) and φ (direction in z, right). Magnitude ranges from 0 (dark blue) to 1.25 (yellow) µm min^{-1}. Color code for φ is as in Figure 4 and ranges from -90 degrees to 90 degrees. Images were acquired every minute and are played back at 6 frames per second. The time stamp shows HH:MM and the scale of the axes grids is 5 µm.

Journal of Cell Science • Supplementary information

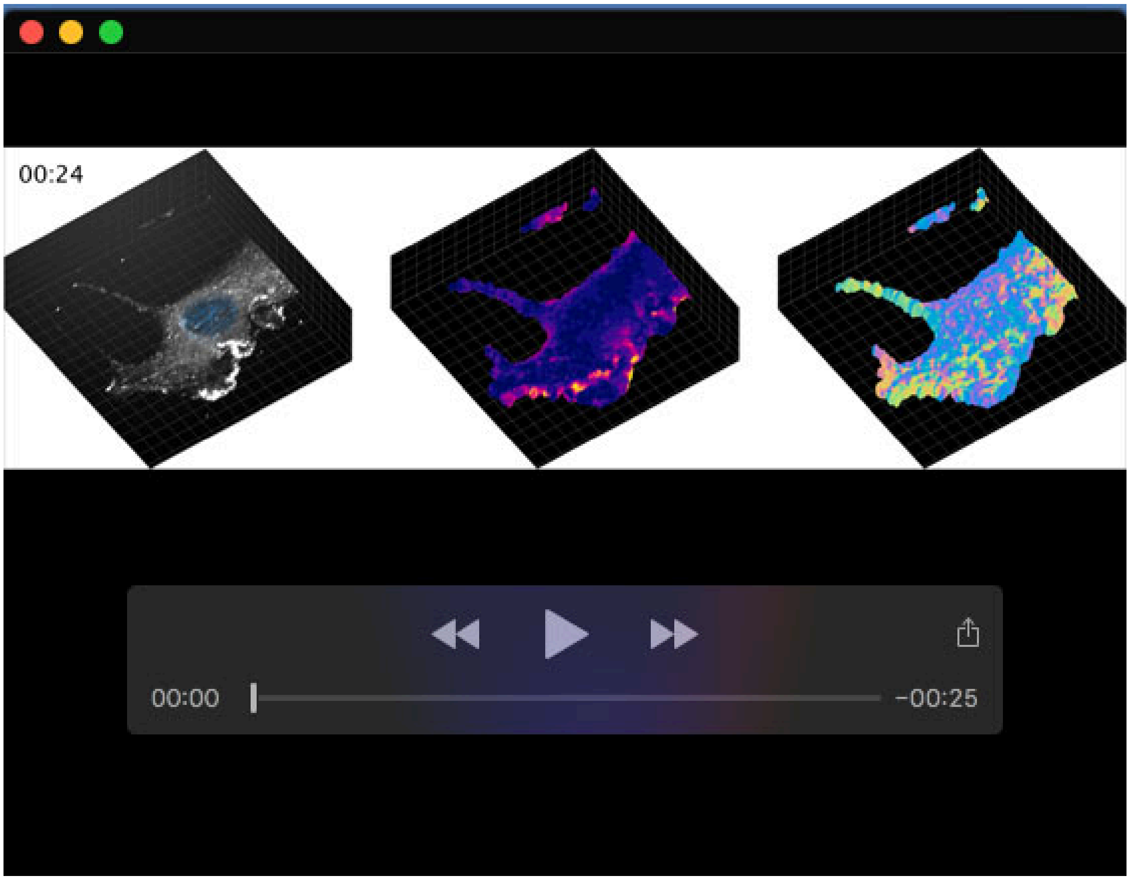

Movie 6. A cropped region of a light sheet image of a migrating cell (left) labeled with Halo-LifeAct (white) and mApple-H2B (blue) is compared to the optical flow magnitude (middle) and ϑ (direction in x and y, right). Magnitude ranges from 0 (dark blue) to 1.25 (yellow) µm min^{-1}. Color code for ϑ is as in Figures 1-4 and ranges from -180 degrees to 180 degrees. Images were acquired every minute and are played back at 6 frames per second. The time stamp shows HH:MM and the scale of the axes grids is 5 µm.

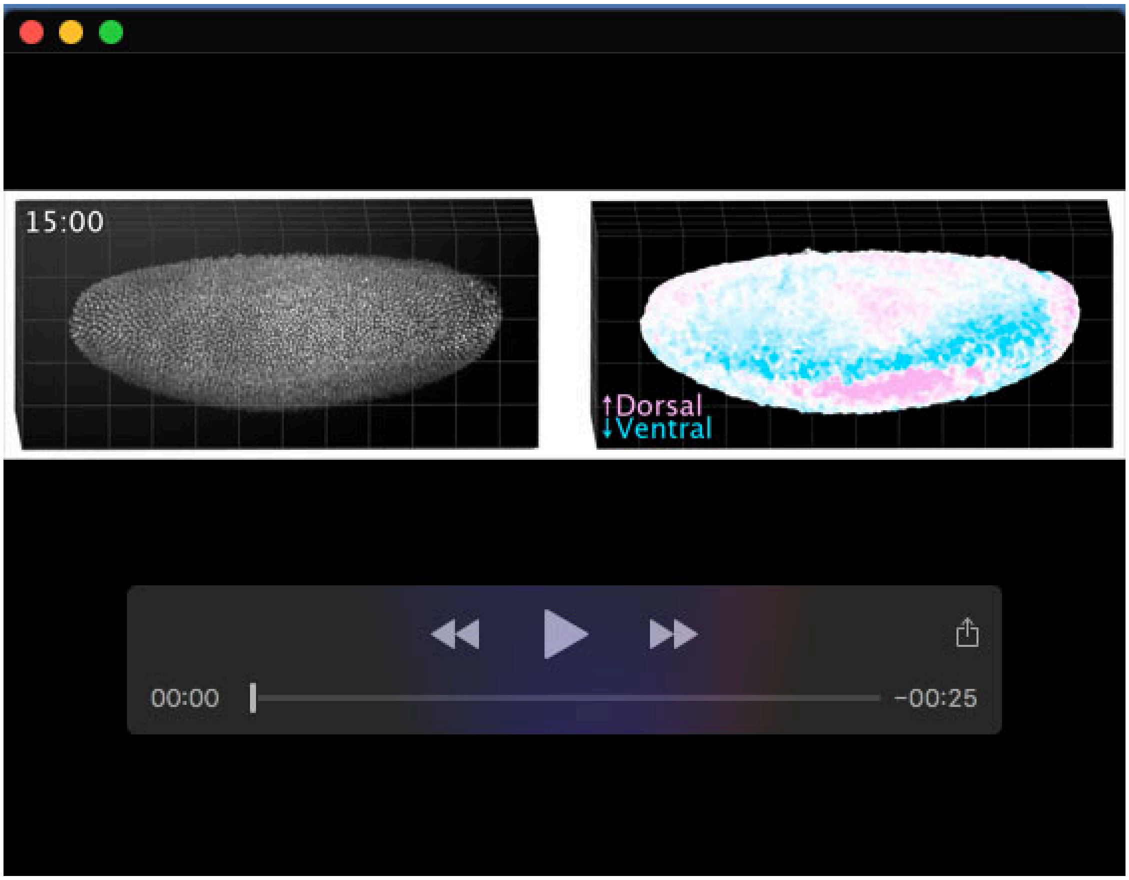

Movie 7. SiMView light sheet imaging of a nuclei-labeled (H2av::mCherry) *Drosophila* embryo (left) is compared to the optical flow field projected on the dorsal-ventral axis (right). The color scale for the flow ranges from -0.75 μm min^{-1} (teal) to 0.75 μm min^{-1} (pink). The time stamp shows MM:SS and the scale of the axes grids is 50 μm.

Journal of Cell Science • Supplementary information

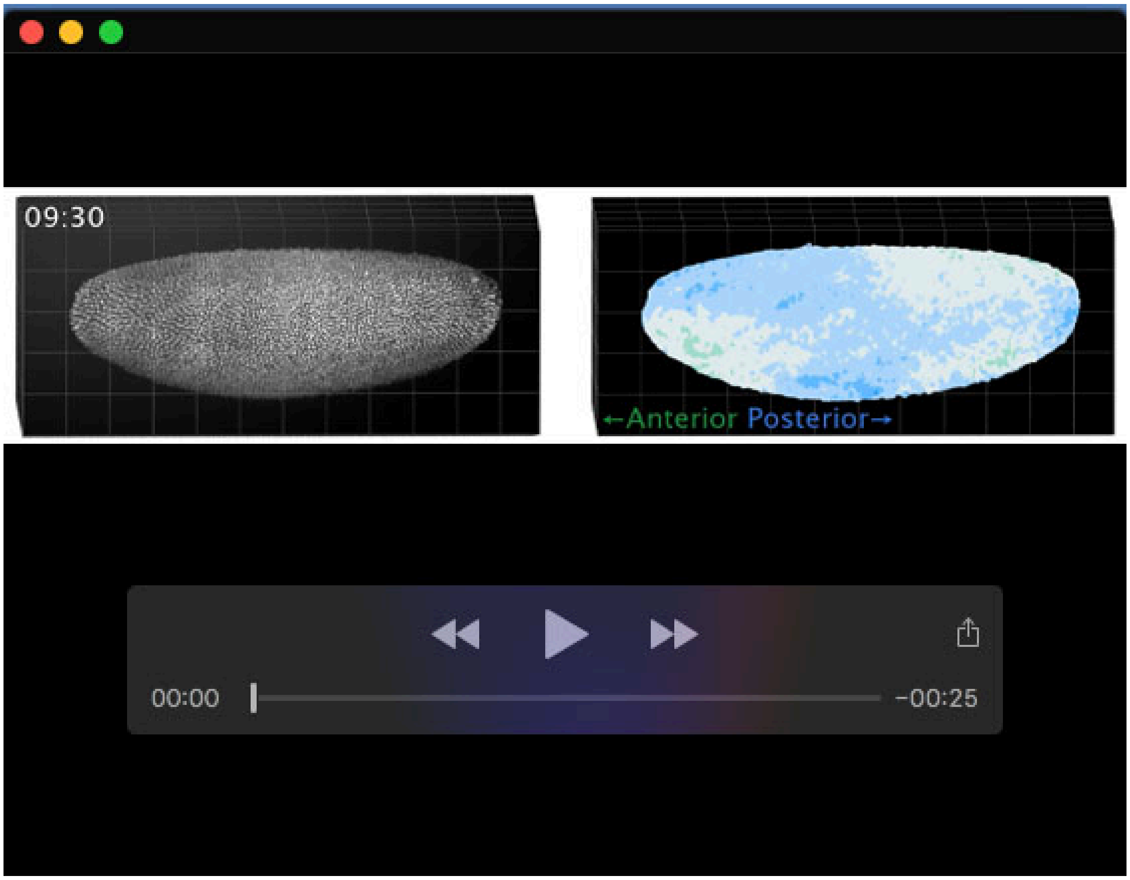

Movie 8. SiMView light sheet imaging of a nuclei-labeled (H2av::mCherry) *Drosophila* embryo (left) is compared to the optical flow field projected on the anterior-posterior axis (right). The color scale for the flow ranges from -2.5 μm min^{-1} (blue) to 2.5 μm min^{-1} (green). The time stamp shows MM:SS and the scale of the axes grids is 50 μm.

Journal of Cell Science • Supplementary information

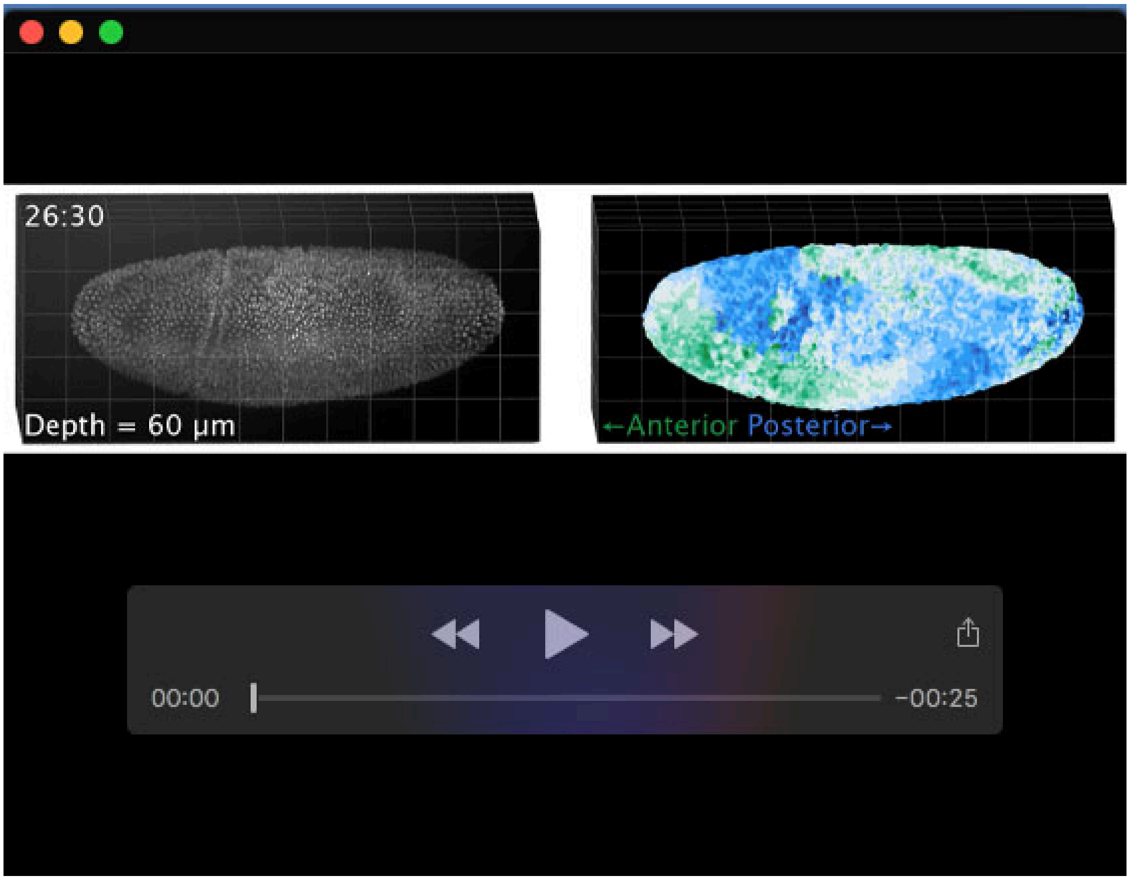

Movie 9. SiMView light sheet imaging of a nuclei-labeled (H2av::mCherry) *Drosophila* embryo (left) is compared to the optical flow field projected on the anterior-posterior axis (right). In the first portion of the movie a single time point (26:30) is shown at cross-sections of increasing z-depth. In the second portion of the movie, both views show a cross section at a depth of 108 µm over time. The color scale for the flow ranges from -2.5 µm min^{-1} (blue) to 2.5 µm min^{-1} (green). The time stamp shows MM:SS and the scale of the axes grids is 50 µm.

Journal of Cell Science • Supplementary information
